# Circular RNAs as diagnostic biomarkers for gastric cancer: A comprehensive update from emerging functions to clinical significances

**DOI:** 10.3389/fgene.2022.1037120

**Published:** 2022-10-28

**Authors:** Chun-Yi Xu, Xi-Xi Zeng, Li-Feng Xu, Ming Liu, Feng Zhang

**Affiliations:** ^1^ Zhejiang Chinese Medical University, Hangzhou, China; ^2^ Core Facility, Quzhou People’s Hospital, The Quzhou Affiliated Hospital of Wenzhou Medical University, Quzhou, China; ^3^ Yangtze Delta Region Institute (Quzhou), University of Electronic Science and Technology of China, Quzhou, China; ^4^ The Joint Innovation Center for Engineering in Medicine, Quzhou, China; ^5^ University of Electronic Science and Technology of China, Chengdu, China

**Keywords:** circular RNA, gastric cancer, biomarker, bodily fluids, diagnosis

## Abstract

The incidence and mortality of gastric cancer ranks as a fouth leading cause of cancer death worldwide, especially in East Asia. Due to the lack of specific early-stage symptoms, the majority of patients in most developing nations are diagnosed at an advanced stage. Therefore, it is urgent to find more sensitive and reliable biomarkers for gastric cancer screening and diagnosis. Circular RNAs (circRNAs), a novel type of RNAs with covalently closed loops, are becoming a latest hot spot in the field of. In recent years, a great deal of research has demonstrated that abnormal expression of circRNAs was associated with the development of gastric cancer, and suggested that circRNA might serve as a potential biomarker for gastric cancer diagnosis. In this review, we summarize the structural characteristics, formation mechanism and biological function of circRNAs, and elucidate research progress and existing problems in early screening of gastric cancer.

## 1 Introduction

Gastric cancer (GC) is one of the most prevalent forms of cancer. It ranks fifth and fourth in morbidity and mortality respectively among all tumors. There are geographical and populational distribution differences in different regions, among which East Asia, South America, Central America and Eastern Europe have higher incidence rates than those of other regions (2020). Particularly in Japan, South Korea, and China, gastric cancer is one of the most commonly diagnosed cancers (Bray et al., 2018). Early-stage gastric cancer lacks specific symptoms, making it difficult to detect. Approximately two-thirds of gastric cancer patients in China are diagnosed at an advanced stage, which lacks effective treatments (Shen et al., 2013). Even given the neoadjuvant therapy combined with surgery, the 5-year progression-free rate in patients with advanced gastric cancer is only 20%–30% (Sitarz et al., 2018). At present, endoscopic biopsy and histopathological examination are the gold standards for gastric cancer diagnosis. However, due to the discomfort caused during gastroscopy, general acceptance of endoscopy by the population as a screening approach remains low. In particular, endoscopy is restrictive in elderly patients and patients with cardiopulmonary insufficiency (Yao 2013). In addition, traditional laboratory tumor markers such as CEA, CA19-9, CA12-5, and CA72-4 have poor sensitivity and specificity in the detection of gastric cancer in the early stage (Sekiguchi and Matsuda 2020). At present, there is an urgent clinical demand for more reliable biomarkers to strengthen the detection of gastric cancer especially in the early stage.

Circular RNAs (circRNAs) are a type of closed-loop non-coding RNA without the 3’end poly-A structure and the 5’end cap structure (Kristensen et al., 2019). In recent years, the rapid development of genome microarray and whole-genome sequencing technology promotes the discovery of novel circRNAs. Research has demonstrated that abnormal expressions of circRNAs are associated with cancer development, and have proposed them as potential biomarkers for cancer diagnosis, including gastric cancer. In this review, we summarize and discuss findings in this field thus far, providing a comprehensive update on the application of circRNAs in the screening and diagnosis of gastric cancer.

## 2 Overview of circular RNAs

### 2.1 The biogenesis and classification of circular RNAs

CircRNAs are molecules of single-stranded RNA that have been covalently closed into a circular structure. Unlike linear RNA, circRNAs lack 5′ to 3′ polarity and polyadenylation [poly(A)] tail. Alternative exon splicing generates linear RNA, whereas circRNAs are typically generated by back splicing the 3′ end of the exon to the upstream exon or the 5′ end of itself. CircRNAs usually contains one to five exons. Therefore, circRNAs are resistant to ribonuclease (RNase) and exonuclease degradation, with a half-life of up to 48 h (Kristensen et al., 2019). The long half-life and tissue-specific expression pattern of circRNAs make them more appealing as diagnostic markers compared to other forms of RNAs.

CircRNAs can be divided into exonic circRNAs (ecircRNAs) formed only by exon sequences, intronic circRNAs (ciRNAs) formed by intron sequences, and exon-intron circular RNAs (EIciRNAs) composed of both exon and intron sequences, depending on their source (Zhu et al., 2019). The circularization process of circRNAs has been intensely studied, and several models have been investigated and validated. 1) Lasso-driven circularization model: the splice donor and splice acceptor form a lasso containing exons connected through covalent bonding, thereby forming ecircRNAs. 2) Intron pair-driven circularization model: complementary bases flanking introns bind together and bring two adjacent exons together. Introns are then removed by the spliceosome. Subsequently the splicing sites are joined to form EIciRNAs or ecircRNAs. 3) Intron circularization model: The remaining lasso introns in the pre-mRNA are circularized by the GU-rich sequence near the 5′ splice site and the C-rich sequence near the branch point. The circularized introns are further cut to form stable ciRNA. This ciRNA, which forms a lasso structure by connecting its two ends, can resist exonuclease degradation and has high stability. These structural characteristics are of great significance in the screening of cancers (Kristensen et al., 2019; Su et al., 2019).

The dynamics of the circularization of circRNAs are influenced by numerous factors, Zhang et al. (2014) showed that exon circularization depended on the complementary sequences of flanking introns, and the efficiency of circularization is controlled by the rivalry between RNA pairing across flanking introns and within individual introns. In addition, it was reported that proteins such as MBL (Muscleblind protein) were involved in the formation of circRNAs. MBL has binding sites on the flanking introns of its pre-mRNA that can promote the circularization of circRNAs (Ashwal-Fluss et al., 2014).

### 2.2 The biological functions of circular RNAs

In recent years, extensive research has been conducted on the biological functions of circRNAs, and several major functions have been elucidated. Firstly, circRNAs can act as competitive inhibitors of miRNA by binding to miRNAs, also known as “miRNA sponges,” or as target mimics to inhibit the activity of a specific miRNA (Hansen et al., 2013). For example, ciRS-7 indirectly up-regulates the expressions of miR-7 target genes by binding to miR-7 and therefore participating in processes such as insulin secretion, myocardial infarction and gastric cancer (GC) progression (Zheng et al., 2017; Pan et al., 2018a). Secondly, circRNAs interact with RNA binding proteins (RBPs) and thus indirectly affect the signaling pathways downstream of RBPs (Du et al., 2017). Thirdly, circRNAs work with U1 snRNP to stimulate the transcription of their parental genes (Li et al., 2015b). A few circRNAs can also function as templates for protein translation (Pan et al., 2018b).

## 3 CircRNAs in gastric cancer

### 3.1 Abnormal expression of circular RNAs in gastric cancer

**FIGURE 1:**
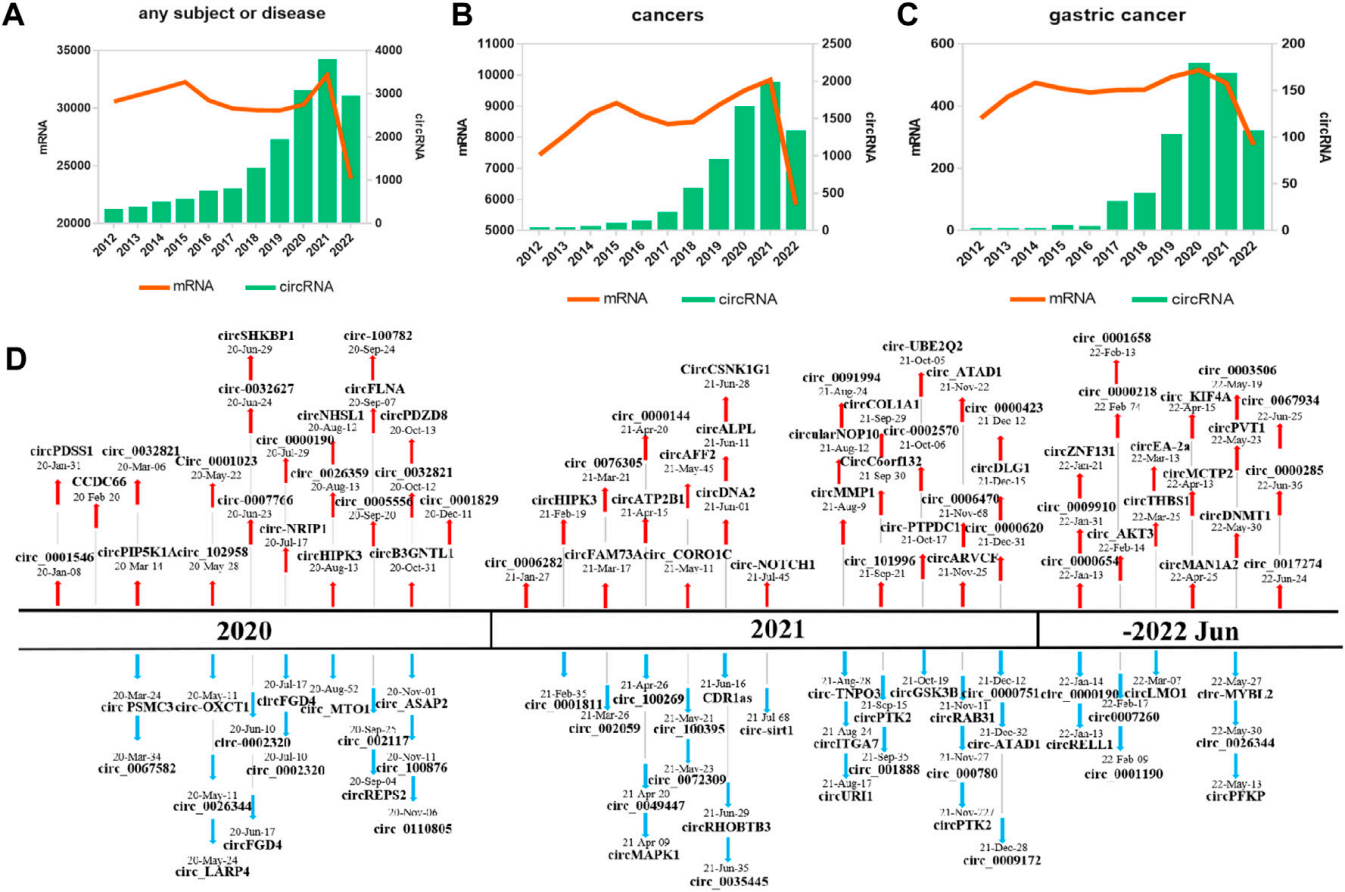
Research on and discovery of circRNAs in GC. The amount of research, as quantified by the annual number of peer-reviewed publications, has been relatively stable for mRNAs (orange line) but not for circRNAs (blue bars) in the following categories: **(A)** an overall, for any subject or disease; **(B)** cancers; **(C)** GC. **(D)** Increasing numbers of novel circRNAs were identified from 2020 to July 2022.

A comprehensive review was conducted by searching PubMed for articles with the keywords (“circular RNA” and “gastric cancer”) published over the past 10 years (January 2012–August 2022). Multiple studies have explored that the discovery and characterization of circRNAs in GC has increased annually, while protein-coding gene (mRNA) discovery research has remained stable (Figures 1A–C). These results show a rising fascination with circRNAs and their involvement in GC. Overall, related studies have validated 115 circRNAs (67 upregulated and 48 downregulated) in the past 3 years (Figure 1D).

**FIGURE 2:**
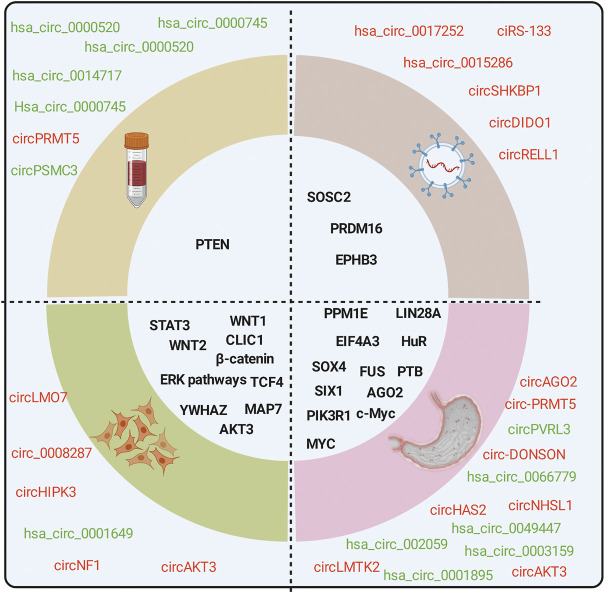
CircRNAs are associated with the hallmarks of GC. CircRNAs are differentially expressed in GC tissues, cells, exosomes, and blood from patients with GC compared with normal controls. Red for upregulation, green for downregulation.

Thousands of circRNAs have been identified by circRNA-specific microarrays and RNA-seq in GC tissues, cells, blood, and exosomes from patients with GC (Figure 2). Most of the gastric cancer-associated circRNAs are expressed in cancer tissues, with only a few circRNAs in body fluids. CircRNAs in plasma is easier to use for disease prediction and therapeutic efficacy judgment due to differences in ease of access in tissues.

#### 3.1.1 Dysregulated circular RNAs in gastric cancer cells

Using high-throughput RNA-seq, Guo et al. (2022a) analyzed circRNA expression profiles in PBS-treated and Helicobacter pylori-infected AGS cells. As compared to the control, among 18,308 different circRNA candidates, the experiment yielded 101 significantly differentially expressed circRNAs, including 84 upregulated and 17 downregulated circRNAs. In addition, numerous studies have reported that circRNAs in gastric cell lines are dysregulated. CircAKT3 was identified as being overexpressed in MKN-7 and HGC-27 cells compared to GES-1 cells (Huang et al., 2019). Consistent with these findings, Yang et al. (2021a) determined that the expression level of circHIPK3 was elevated in gastric cancer cell lines compared with normal gastric cell lines. In addition, the expression of circLMO7 was significantly higher in gastric cancer cells than in GES-1 cells (Cao et al., 2021). These studies suggest that circRNA promotes the progression of gastric cancer.

#### 3.1.2 Dysregulated circular RNAs in gastric cancer tissues

To identify the circRNAs involved in GC tumorigenesis, a recent study detected differential circRNA expression between GC tissues and adjacent noncancerous tissues. In a study by Shao et al. (2017), among the 308 significantly differentially expressed circRNAs, there were 107 (34.74%) upregulated ones. However, the majority (65.26%) of circRNAs were found to be down-regulated in cancer tissues. In addition, Zhang et al. (2017b) detected 3,071 expressed circRNA indicators in the six pairs of tumors and adjacent normal mucosal specimens, among these circRNAs, 46 indicators revealed different expression levels. Another study performed large-scale gene screening in three pairs of GC tissues using high-throughput sequencing, 25,303 circRNAs were detected in the screening. Of these circRNAs, 2,007 DECs were identified based on the filter criteria of |FC| ≥ 2, *p* < 0.05 (Kong et al., 2019). Based on RNA-seq, Jie et al. (2020) found most of these circRNAs originated from exons, And thirteen candidate circRNAs were significantly downregulated and 9 were upregulated which were analyzed by ggplot2 between 30 pairs of gastric cancer and adjacent normal cancer tissues. Wang et al. (2021b) applied ribosomal RNA (rRNA)-depleted RNA-seq analysis of five-paired GC and normal tissues to systematically characterize the genome-wide landscape of circRNAs in GC. The result displayed 4485 circRNAs in GC and 5008 circRNAs in normal tissue. Among the dysregulated circRNAs, 245 candidates were significantly dysregulated (152 downregulated and 93 upregulated) in GC.

These sequencing and bioinformatics analysis illustrate the dysregulation of circRNA profiles in GC, However, the precise role and internal mechanisms of circRNAs in GC remain elusive.

#### 3.1.3 Dysregulated circular RNAs in blood from patients with gastric cancer

Liquid biopsy is a noninvasive technique that utilizes body fluids such as blood, urine, and gastric juice to determine the disease state (Reimers et al., 2019). Identifying circulating tumor markers in blood and other bodily fluids has been one of the research focuses in this area (Batth et al., 2017). Recently, circular RNAs (circRNAs) have attracted considerable attention in tumor biopsies as detection and quantitative biomarker (de Fraipont et al., 2019). Although research on circRNAs is in its infancy, numerous studies have indicated their potential as useful biomarkers for the diagnosis and prognosis of cancer (Arnaiz et al., 2019).

For instance, increased expression of serum circSHKBP1 (hsa_circ_0000936) level was significantly associated with poor survival and advanced TNM stage (Xie et al., 2020). CircPSMC3 was downregulated in plasmas in GC patients. Lower circPSMC3 expression was associated with a higher TNM stage and shorter overall survival in GC patients (Rong et al., 2019). It was discovered that hsa_circ_0000520 was significantly down-regulated in gastric cancer plasm compared to normal control. The hsa_circ_0000520 plasma concentration was linked to CEA expression based on clinicopathological characteristics. (Sun et al., 2018). Also, hsa_circ_0000745 was downregulated in GC plasma samples compared with healthy controls (*p* < 0.001). The plasma hsa_circ_0000745 levels were correlated with the stage of tumor-node metastasis. And the AUC of plasma hsa_circ_0000745 was elevated in conjunction with the level of carcinoembryonic antigen (CEA), which suggests plasma hsa_circ_0000745 is a good diagnostic biomarker (Huang et al., 2017). Furthermore, the group reported that hsa_circ_0000181 levels in plasma from GC patients were significantly lower than those in adjacent non-tumorous tissues and in healthy individuals (*p* < 0.001). In addition, the sensitivity of plasma hsa_circ_0000181 were 85.2% and 99.0% respectively (Zhao et al., 2018). A previous study demonstrated that hsa_circ_0000211, hsa_circ_0000284 and hsa_circ_0004771 exhibited identical expression profiles when analyzed by distinct techniques (RNA-Seq and RT-qPCR) and distinct sample types (tissue and blood) (Reis-das-Mercês et al., 2022).

At present, traditional circulating tumor markers in the setting of clinical laboratories such as CEA and CA19-9 have low specificity and sensitivity (Sekiguchi and Matsuda 2020), which limited their clinical application. On the other hand, studies have confirmed that circRNAs exist not only in tissues but also in human serum, plasma and other bodily fluids, especially enriched in microvesicles and exosomes (Li et al., 2015a). Therefore, circRNAs have the potential to be candidates as non-invasive tumor markers.

#### 3.1.4 Dysregulated circRNAs in gastric cancer exosomes

Exosomes are nano-sized vesicles secreted by various cells that express exosome markers such as TSG101, HSP70, CD9, and CD63 but not albumin or calnexin (Feng et al., 2019; Hon et al., 2019). Transmission electron microscopy (TEM) images of exosomes typically depict translucent cup-shaped or spherical structures with diameters ranging from 30 to 150 nm (Xu et al., 2018; Mathieu et al., 2019). In recent years, it has been discovered that exosomes transport miRNAs, lncRNAs, proteins, and even circRNAs for intercellular signal transduction (Xu et al., 2018; Mathieu et al., 2019).

Systematic administration of circDIDO1 through exosome-mediated gene suppressed the tumorigenicity and aggressiveness of GC *in vitro* and *in vivo*, indicating that RGD-Exo-circDIDO1 could be employed as a nanomedicine for the treatment of GC (Guo et al., 2022b). In addition, GC cells’ exosomal hsa_circ_0017252 inhibited GC progression by inhibiting macrophage M2-like polarization. These findings enhance our fundamental comprehension of GC and suggest a novel strategy for developing more effective GC treatments (Song et al., 2022). The expression level of exosomal hsa_circ_0015286 decreased significantly in GC patients following surgery. Patients with low hsa_circ_0015286 expression had a longer overall survival than those with high expression. Exosomal hsa_circ_0015286 may be a promising noninvasive biomarker for the diagnosis and prognostic evaluation of GC (Zheng et al., 2022). CircRELL1 is transmissible *via* exosomal communication, and exosomal circRELL1 inhibited the malignant behavior of GC *in vivo* and *in vitro*. This work reveals a promising novel circulating diagnostic biomarker and treatment target for GC (Sang et al., 2022). These circRNAs may play regulatory functions in the start of GC and may serve as biomarkers for the diagnosis of GC in liquid biopsies.

### 3.2 Molecular mechanisms of circular RNAs in gastric cancer

In an authoritative review, Kristensen et al. (2019) summarized that circRNAs perform regulatory roles may exert their biological functions by acting as miRNA sponges or decoys, protein sponges or decoys, enhancers of protein function, protein scaffolding, protein recruitment and templates for translation. The majority of circRNAs serve as microRNA (miRNA) sponges or decoys, shielding target mRNAs from miRNA-dependent destruction, thus inhibiting the activities of the corresponding miRNAs. The circRNAs that show different biological functions in GC are summarized in Table 1.

**TABLE 1:** CircRNAs associated with human gastric cancer.

CircRNAs	Deregulation	Mechanism (target genes)	Functions	References
circPVT1	Increased	miR-125	Cell growth	Chen et al. (2017)
circLMTK2	Increased	miR-150-5p	Cell growth and metastasis	Wang et al. (2019a)
circAGO2	Increased	miR-224-5p, miR-143-3p	Cell growth, invasion, and metastasis	Chen et al. (2019b)
circ-DONSON	Increased	SOX4	Cell growth and invasion	Ding et al. (2019)
circFNDC3B	Increased	E-cadherin, CD44	Cell migration and invasion	Hong et al. (2019)
circAKT3	Increased	miR-198, PIK3R1	Cell growth and apoptosis	Huang et al. (2019)
circRBMS3	Increased	miR-153, SNAI1	Cell growth and invasion	Li et al. (2019)
circPDSS1	Increased	miR-186-5p, NEK2	Cell cycle and apoptosis	Ouyang et al. (2019)
circNF1	Increased	miR-16	Cell growth	Wang et al. (2019c)
ciRS-133	Increased	miR-133	White adipose tissue browning, cancer-associated cachexia	Zhang et al. (2019a)
circDLST	Increased	miR-502-3p, NRAS/MEK1/ERK1/2	Cell viability, invasion, and metastasis	Zhang et al. (2019b)
circCACTIN	Increased	miR-331-3p, TGFBR1	Cell growth and metastasis	Zhang et al. (2019c)
circNRIP1	Increased	miR-149-5p	Cell growth and metastasis	Zhang et al. (2019d)
circNHSL1	Increased	miR-1306-3p	Cancer invasion and metastasis	Zhu et al. (2019)
circSERPINE2	Increased	miR-375, YWHAZ	Cell growth	Liu et al. (2019)
circHIPK3	Increased	miR-637, AKT1	Cell growth	Yang et al. (2021a)
circPRMT5	Increased	miR-145, miR-1304	Cell growth and metastasis	Du et al. (2019)
circSFMBT2	Increased	miR-182-5p	Cell growth	Li et al. (2020)
hsa_circ_0078607	Increased	miR-188-3p	Cell growth	Bian et al. (2021)
circSMAD4	Increased	miR-1276, CTNNB1	Cell growth	Wang et al. (2021a)
circLMO7	Increased	miR-30a-3p, WNT2/β-Catenin	Cell growth and metastasis	Cao et al. (2021)
circDUSP16	Increased	miR-145-5p	Cell growth and invasion	Zhang et al. (2020)
circOSBPL10	Increased	miR-136-5p, WNT2	Cell growth and metastasis	Wang et al. (2019b)
circSHKBP1	Increased	miR-582-3p, HUR/VEGF	Cell growth and metastasis	Xie et al. (2020)
circHECTD1	Increased	miR-137, PBX3	Cell growth	Lu et al. (2021)
hsa_circ_0081143	Increased	miR-646, CDK6	Cell growth and invasion	Lu et al. (2021)
circHAS2	Increased	miR-944, PPM1E	Cell growth and invasion	Ma et al. (2021a)
hsa_circ_0000993	Decreased	miR-214-5p	Cell growth and metastasis	Zhong et al. (2018)
circHuR	Decreased	HuR	Cell growth and invasion	Yang et al. (2019)
circHIAT1	Decreased	miR-21	Cell growth and migration	Quan et al. (2020)
circLARP4	Decreased	miR-424, LATS1	Cell growth and invasion	Zhang et al. (2017a)
circCUL2	Decreased	mir-142-3p, VAMP3	Cell growth and metastasis	Peng et al. (2020)
circPSMC3	Decreased	miR-296-5p	Cell growth and migration	Rong et al. (2019)
circRNA_100,269	Decreased	miR-630	Cell growth	Zhang et al. (2017c)
circYAP1	Decreased	miR-367-5p	Cell growth and invasion	Liu et al. (2018)
circFAT1(e2)	Decreased	miR-548g, RUNX1	Cell growth and metastasis	Fang et al. (2019)
circMCTP2	Decreased	miR-99a-5p, MTMR3	Cell proliferation and apoptosis	Sun et al. (2020)
circREPS2	Decreased	miR-558, RUNX3/β-catenin	Cell growth and migration	Guo et al. (2020)
circCCT3	Decreased	miR-613, VEGFA/VEGFR2	Cell migration and invasion	Hou et al. (2021)
circCCDC9	Decreased	miR-6792-3p, CAV1	Cell growth	Luo et al. (2020)
circSPECC1	Decreased	miR-526b, KDM4A/YAP1	Cell growth and invasion	Chen et al. (2019a)
circMRPS35	Decreased	KAT7/FOXO1/3a	Cell growth and invasion	Jie et al. (2020)
circRPPH1	Decreased	miR-512-5p, STAT1	Cell growth	Huang et al. (2021)
circRHOBTB3	Decreased	miR-654-3p, p21	Cell growth	Deng et al. (2020)
circMAPK1	Decreased	MAPK1	Cell growth and invasion	Jiang et al. (2021)

CircNHSL1 acts as a sponge for miR-1306-3p to alleviate its suppression of SIX1 target. Enhanced expression of circNHSL1 promotes invasion and metastasis of gastric cancer (Zhu et al., 2019). Functionally, circPVT1 serves as a sponge for miR-125 family members to stimulate cell proliferation (Chen J et al., 2017). Overexpression of circLMTK2 enhances gastric cell proliferation, migration and invasion *in vitro* and *in vivo*. CircLMTK2 absorbs miR-150-5p and then indirectly regulates the expression of c-Myc to promote gastric cancer carcinogenesis (Wang et al., 2019a). *In vitro* and *in vivo* studies indicate that circAGO2 enhances the development, invasion, and dissemination of gastric cancer cells. Mechanistic studies demonstrate that circAGO2 physically interacts with the human antigen R (HuR) protein to assist the HuR-repressed actions of AGO2-miRNA complexes that promote cancer progression (Chen et al., 2019b). The silencing of circDONSON substantially inhibited GC cell proliferation, migration, and invasion, while promoting apoptosis. Functionally, circDONSON recruits the NURF complex to the promoter of SOX4 and initiates its transcription to facilitate gastric cancer growth and metastasis (Ding et al., 2019). CircPDSS1 enhanced GC cell cycle and reduced apoptosis by preventing miR-186-5p from targeting NEK2 to promote apoptosis. Therefore, circPDSS1 may serve as a biomarker and therapeutic target for the treatment of GC (Ouyang et al., 2019).

GSPT1-238aa, a novel protein encoded by circGSPT1, was discovered as a selective translation driven by IRES. GSPT1-238aa modulates autophagy can interact with vimentin/Beclin1/14-3-3 complex *via* the PI3K/AKT/mTOR signaling pathway in GC cells (Hu et al., 2022). What’s more, AXIN1-295aa as a novel protein encoded by circAXIN1, it functions as an oncogenic protein to promote GC tumorigenesis and progression by activating the Wnt signaling pathway, suggesting a potential therapeutic target for GC (Peng et al., 2021).

Another study revealed that circST3GAL6 controlled apoptosis and autophagy *via* FOXP2-mediated transcriptional regulation of the MET axis *via* the miR-300/FOXP2 axis, which may represent a viable GC treatment target (Xu et al., 2022b). Ebv-circRPMS1 binds to Sam68 to promote its physical contact with the METTL3 promotor, resulting in transactivation of METTL3 and development of cancer (Zhang et al., 2022b). Ebv-circLMP2A interacted with KHSRP to increase the KHSRP-mediated degradation of VHL mRNA, resulting in an accumulation of HIF1 under hypoxia, which was crucial in controlling tumor angiogenesis in EBVaGC and might be a good therapeutic target for EBVaGC (Du et al., 2022). Circ-TNPO3 can bind competitively with IGF2BP3 and reduce IGF2BP3’s capacity to stabilize MYC mRNA, ultimately inhibiting the proliferation and metastasis of GC (Yu et al., 2021).

Most of the circRNAs were located in the cytoplasm, However, circGSK3B was mainly identified in the nucleus. CircGSK3B is able to interact directly with EZH2, inhibiting the binding of EZH2 and H3K27me3 to the RORA promoter (Ma et al., 2021b).

### 3.3 Biological functions of circular RNAs in gastric cancer

#### 3.3.1 The functions of circular RNAs in gastric cancer: Based on *in vivo* evidence

Due to the large number of circRNAs studied, a large number of circRNAs are reported every year for the role in gastric cancer. Nevertheless, most of these studies are based on the data from *in vitro* cell culture. *In vivo* investigation provides much more In-depth perspectives for these circRNAs. In particular, we summarize here relevant studies of circRNAs with relatively well-established functional studies *in vivo* in gastric cancer to help us understand which circRNAs functions have received focused attention and a more comprehensive understanding.

Numerous factors contribute to the biological makeup of GC. A recent study demonstrated that HOTAIR upregulation was associated with shorter overall survival in patients with gastric cancer, as well as advanced pathological stage, larger tumor size, and extensive metastasis. In addition, HOTAIR overexpression promoted the progression of gastric carcinoma *in vitro* and *in vivo via* regulating HER2 expression as a ceRNA of miR-331-3p (Liu et al., 2014). Furthermore, circAKT3 (hsa_circ_0000096) was significantly downregulated in gastric cancer tissues relative to nearby nontumorous tissues and normal gastric epithelial cells (*p* < 0.001). CircAKT3 might stimulate PIK3R1 expression *via* sponging miR-198, thereby increasing DNA damage repair and preventing apoptosis *in vivo* and *in vitro* in GC cells (Huang et al., 2019). The knockdown of hsa_circ_0000096 markedly decreased cell proliferation and migration *in vivo* (Li et al., 2017a). In GC tissues and cells, the amount of circCUL2, which is stable and restricted to the cytoplasm, was drastically decreased. Overexpression of circCUL2 decreased *in vivo* tumorigenicity (Peng et al., 2020).

#### 3.3.2 The role of circular RNAs in gastric cancer *in vitro*

Silencing circRBMS3 decreased GC cell proliferation and invasion through sponging miR-153 *in vitro* (Li et al., 2019). Loss- and gain-of-function experiments indicate that circNF1 greatly increases GC cell proliferation (Wang et al., 2019c). Moreover, circCACTIN could function as a sponge of miRNA-331-3p and modulate the mRNA expression of TGFBR1. Knockdown of circCACTIN reduced the capability of cells proliferation, migration and invasion in GC cells (Zhang et al., 2019c).

## 4 The clinical values of circular RNAs in gastric cancer

As alluded to earlier, many circRNAs do not have high sensitivity on their own, however, the combination of these circRNAs with other tumor markers or circRNAs can dramatically improve the sensitivity and specificity in early gastric cancer screening. For example, the combined detection of hsa_circ_0001017 and hsa_circ_0061276 in gastric cancer tissues and patient plasma has a high diagnostic value, with AUC as high as 0.966, and sensitivity and specificity of 95.5% and 95.7%, respectively (Li et al., 2018). The independent AUCs of hsa_circ_0000096 and hsa_circ_002059, both downregulated in gastric cancer tissue, were 0.82 and 0.73, but the AUC increased to 0.91 when these two circRNAs were used in combination (Li et al., 2017a). In addition, hsa_circ_0000745 was reduced in gastric cancer patients’ plasma with an AUC of merely 0.683, but when hsa_circ_0000745 was combined with CEA, the AUC rose dramatically to 0.775 (Huang et al., 2017). These studies revealed that combining multiple circRNAs or with traditional diagnostic markers such as CEA, and CA19-9 can increase the sensitivity, specificity and accuracy of circRNAs based on gastric cancer diagnosis and prognosis. It provides new perspectives for developing circRNAs as diagnostic markers for early gastric cancer screening.

Although early-stage gastric cancer is highly curable through surgery, the majority of patients are diagnosed at an advanced stage, and therefore missed the window of opportunity for surgery. The high incidence and high mortality of gastric cancer urgently call for an early screening program. Detecting biomarkers in bodily fluids is a patient-friendly approach as bodily fluids are easy to obtain with likely higher patient compliance than endoscopy. The aforementioned studies demonstrated the potential of circRNAs as diagnostic markers for gastric cancer. Firstly, circRNAs have advantages compared with linear RNAs such as stable expression and a high degree of conservation. Secondly, an array of circRNAs is closely associated with gastric cancer development and risk factors, and their abnormal expression can signal tumor development and thus be used as screening biomarkers. Thirdly, in addition to their presence in tissues, circRNAs can also be sampled non-invasively in plasma and other bodily fluids. Lastly, with the development of technology, the detection of circRNAs will become more sensitive and cost-effective.

### 4.1 Diagnostic biomarkers

CA72-4 is currently the standard biomarker for early diagnosis of GC; however, its sensitivity and specificity are not optimal. CircRNAs exhibit distinct expression patterns in the tumor tissues and blood of patients with GC versus those of healthy controls. Thus, they are regarded as promising biomarkers for tissue or liquid biopsies in the diagnosis of GC.

Xie et al. (2018) found that the expression of hsa_circ_0074362 was downregulated in both gastritis and gastric cancer tissues. Due to the association between gastritis and a high risk of gastric cancer, hsa_circ_0074362 was proposed to be an early indication of gastric cancer. Albeit circ_0074362 was not able to function as an independent diagnostic marker of gastric cancer since it has a relatively low sensitivity (0.362), the level of hsa_circ_0074362 was associated with the serum tumor biomarker CA19-9 and lymph node metastasis. Therefore, by combining with other clinical markers, hsa_circ_0074362 may still hold the potential for gastric cancer screening. Another circRNA with a potential diagnostic value is hsa_circ_0001649, which is down-regulated in GC tissues. The ROC curve showed a sensitivity and specificity of 71.1% and 81.6% respectively, and the AUC was 0.834. These findings suggested that hsa_circ_0001649 could be used as a highly accurate, specific, and sensitive biomarker for gastric cancer (Li et al., 2017b).

Perhaps more interesting was the finding that the expression level of exosomal hsa_circ_0015286 decreased significantly in GC patients following surgery, suggesting that exosomal hsa_circ_0015286 may be a promising noninvasive biomarker for the diagnosis and prognostic evaluation of GC (Zheng et al., 2022). Likewise, in another study, a panel of 8 circRNAs as non-invasive, liquid-biopsy biomarkers that could serve as possible diagnostic biomarkers for the early diagnosis of GC were identified (Roy et al., 2022).

### 4.2 Prognostic biomarkers

**FIGURE 3:**
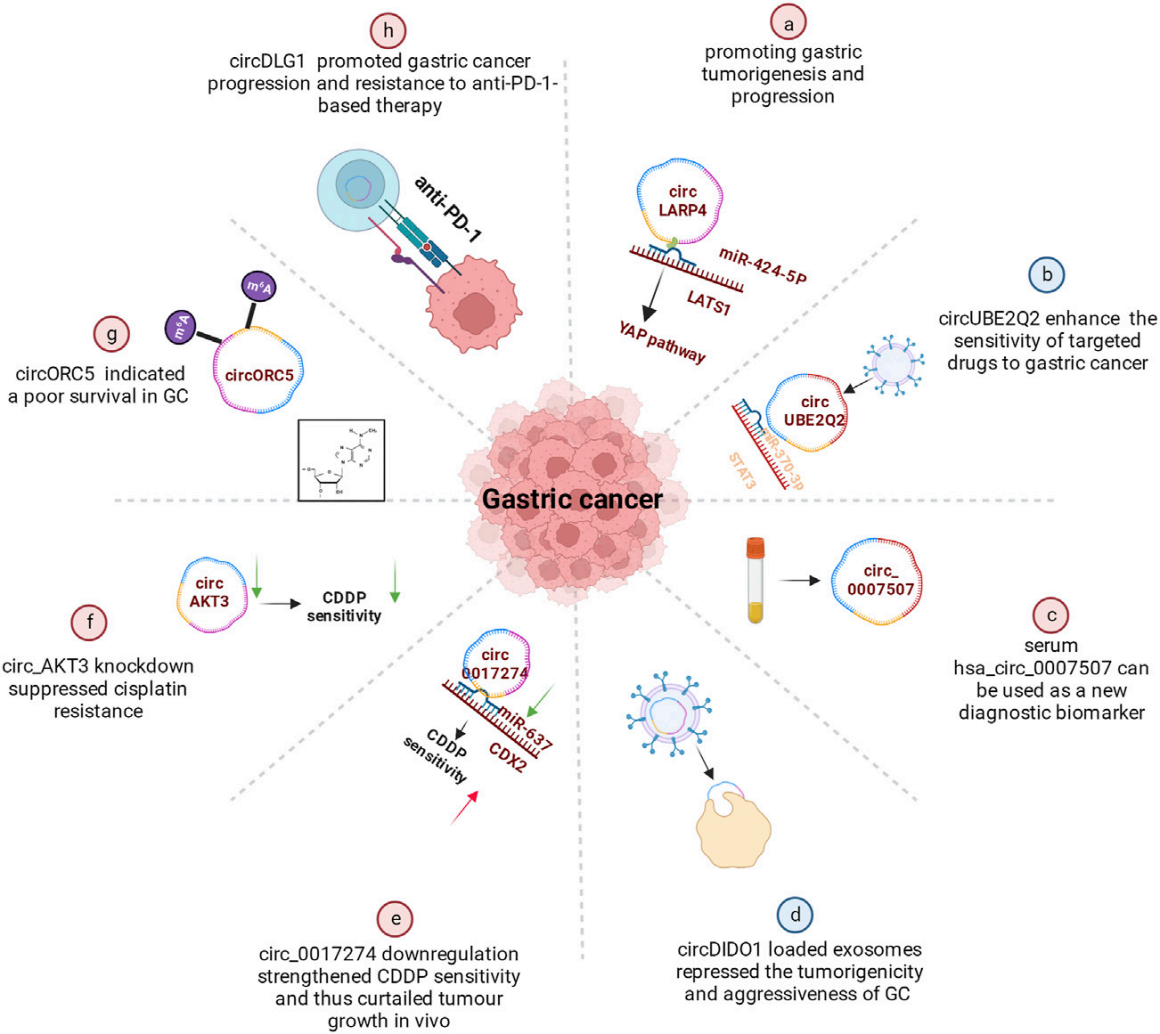
Functional roles of circRNAs in GC. **(A)** CircRNAs promoting gastric tumorigenesis and progression (e.g., circLARP4). A few circRNAs also regulated CDDP resistance **(B)**, cisplatin resistance **(C)** or promoted gastric cancer progression and resistance to anti-PD-1-based therapy **(D)**. **(E)** Some circRNAs indicated a poor survival in GC (e.g., circORC5). **(F)** Serum hsa_circ_0007507 can be used as a new diagnostic biomarker. **(G)** Certain circRNAs could enhance the sensitivity of targeted drugs to gastric cancer (e.g., circUBE2Q2). **(H)** circDIDO1 could repressed the tumorigenicity.

Secondary prevention, including early identification, early diagnosis, and early treatment, can improve GC patients’ prognosis. CircRNAs have been increasingly recognized as potential biomarkers for prognosis. CircLARP4, for example, was mostly located in the cytoplasm and regulated the biological behaviors of GC cells by sponging miR-424. Meanwhile, the decreased expression of circLARP4 in GC tissues was an independent predictive factor for the overall survival of GC patients (Zhang et al., 2017a) (Figure 3A). Further *in vivo* investigations verified that the combination treatment of circUBE2Q2 knockdown and STAT3 inhibitor had synergistic effects on the inhibition of gastric cancer growth, suggesting that targeting circUBE2Q2 may increase the sensitivity of targeted therapies to gastric cancer (Yang et al., 2021b) (Figure 3B). In addition, the differential expression of serum hsa_circ_0007507 among GC, post-operative GC, gastritis, intestinal metaplasia and relapsed patients, suggests it would be useful as a new diagnostic and dynamic monitoring biomarker for GC (Zhang et al., 2021) (Figure 3C). More specifically, the study of Li et al. (2017a) showed that hsa_circ_0000096 was significantly downregulated in gastric cancer tissues (*p* < 0.001) and the AUC was as high as 0.82, indicating high diagnostic accuracy.

### 4.3 Therapeutic targets

A bunch of researches have revealed the relevance of circRNAs in GC and their link with GC carcinogenesis and development, and indicated that circRNAs have the potential to act as therapeutic targets in GC.

For instance, the overexpression of circAKT3 in GC patients undergoing cisplatin (CDDP) therapy was substantially linked with aggressive features and constituted an independent risk factor for disease-free survival (DFS). circAKT3 was expressed at a higher level in CDDP-resistant GC tissues and cells than in CDDP-sensitive samples. Clinicopathological characteristics demonstrated that the level of hsa_circ_0000520 in GC tissues was adversely correlated with TNM stage and that the amount of CEA expression in GC plasma was correlated with TNM stage (Sun et al., 2018).

Another finding showed that circDIDO1 inhibited the advancement of GC through regulating the miR-1307-3p/SOSC2 axis (Figure 3D). Systemic injection of RGD-modified, circDIDO1-loaded exosomes inhibited the tumorigenicity and aggressiveness of GC *in vitro* and *in vivo*, indicating that RGD-Exo-circDIDO1 could be employed as a nanomedicine for the treatment of GC (Guo et al., 2022b).

Hu et al. (2022) confirmed that GSPT1-238aa, a new protein encoded by circGSPT1, inhibits the development of GC tumors. They also shed light on the function and molecular mechanisms behind GSPT1-238aa in GC and suggest that this protein constitutes a unique therapeutic target for GC. Moreover, another circRNA, Circ-MTO1, correlates with less lymph node metastasis, prolonged DFS, and improved chemotherapy sensitivity in gastric cancer (Chang et al., 2022).

### 4.4 Drug resistance

Currently, circular RNAs in significant numbers are now linked to the emergence of treatment resistance and the onset of cancers. By regulating the miR-383-5p/FGF7 axis, knockdown of circLRCH3 reduced GC OXA resistance, providing a prospective therapeutic target for GC chemoresistance (Xiang et al., 2022). As shown in a study by Xu et al. (2022a), circ0017274 was upregulated in GC tissues and cells resistant to CDDP, while miR-637 was lower (Figure 3E). Reducing the abundance of circ_0017274 not only alleviated CDDP resistance but also induced cell cycle arrest in GC cells. The xenograft models further demonstrated that circ0017274 downregulation increased CDDP sensitivity and consequently inhibited *in vivo* tumor growth. By acting on miR-637/CDX2 in CDDP-resistant GC cells, circ0017274 downregulation improved CDDP sensitivity.

It was reported that ICA decreased GC cell survival and induced pyroptosis by modulating the hsa_circ_0003159/miR-223-3p/NLRP3 axis both *in vitro* and *in vivo*. ICA suppresses the proliferation of GC cells *via* modulating the hsa_circ_0003159/miR-223-3p/NLRP3 signalling pathway (Zhang et al., 2022a). Circ_AKT3 knockdown decreased cisplatin resistance in cisplatin-resistant GC cells *via* the miR-206/PTPN14 axis (Shi and Wang 2022) (Figure 3F). Furthermore, the METTL14-mediated m6A alteration of circORC5 inhibits the progression of gastric cancer by modulating the miR-30c-2-3p/AKT1S1 axis (Fan et al., 2022) (Figure 3G). In addition, through targeting PRKAA2, circCPM plays a vital role in regulating GC autophagy and 5-FU resistance. It could serve as a novel theoretical foundation for evaluating the therapeutic efficacy of GC and reversing 5-FU chemoresistance (Fang et al., 2022). CircDLG1 was highly increased in distant metastatic lesions and anti-PD-1-resistant gastric cancer tissues, and was linked with an aggressive tumor phenotype and poor prognosis in gastric cancer patients treated with anti-PD-1 drugs (Chen et al., 2021) (Figure 3H).

On the basis of this mounting evidence, circRNAs play increasingly crucial roles in the regulation of drug development.

## 5 Perspectives

### 5.1 Insights and limitations of current research

The clinical application of circRNAs has broad prospects, but it still faces many difficulties. First of all, the cost of circRNA testing is still higher than that of existing gastroscopy testing, which limits its application at the population-scale for early gastric cancer screening. Secondly, the research on circRNAs is still in its infancy, and the diagnostic accuracy and consistency are less than optimal. Current research shows that the sensitivity, specificity and diagnostic accuracy of different circRNAs are highly variable, and gastroscopic pathological biopsy is still the “gold standard” for clinical diagnosis of gastric cancer. Therefore, it still needs more research to screen out the most efficient circRNA candidates and study their values in combination with traditional tumor markers to achieve the best diagnostic results. Furthermore, current research on circRNAs focuses on tissue and blood samples as the source, and the research on circRNAs in other types of bodily fluids is scarce. Since circRNAs are abundant and stably expressed in other types of bodily fluids (Pardini et al., 2019), they should be explored further in the future. Overall, there is still a long way to go until we can establish circRNAs as non-invasive tumor markers in the clinical settings.

### 5.2 Innovative suggestions for future research

In recent years, a growing number of studies have uncovered fundamental aspects of circRNAs and produced many surprising results indicating that circRNAs are important in biology and pathobiology; consequently, circRNA-related research is advancing at a constant and rapid rate. Nonetheless, a global and exhaustive understanding of circRNAs associated with GC early detection is still lacking.

In the section that follows, we propose a number of innovative and challenging directions in the field of circRNAs in future. First, the majority of current investigation is still carried out in cells and animals, how to facilitate translation toward clinical application would be a hot topic in the future. Second, circRNAs as a stable and can be widely detected in many types of body fluids, it is urged to confirm their potential as novel drugs, therapeutic targets, or biomarkers. In addition, Whether or not a circRNA with a low abundance can achieve measurable effects remains debatable. Unlike most current studies that explore the effect of a single circRNA on a specific physiological process, the investigation in future should focus on a group of circRNAs with similar functions that affect the physiological processes.

## 6 Summary and outlook

As a newly discovered type of RNA molecule, circRNAs have important biological functions and the potential to become an early biomarker for gastric cancer screening. The volume of research on this topic has been steadily growing in the past several years. Multiple studies have revealed the potential of circRNAs as biomarkers for gastric cancer as they are highly conservative and differentially expressed in gastric cancer patients, while the current research is still limited in scope. The interactions between circRNAs, miRNAs and RBPs and the mechanisms underlying their functions in gastric cancer are not yet fully understood. However, with these mechanistic questions being studied and answered, circRNAs will likely to become a novel marker in the early screening of gastric cancer to improve the survival rate of patients.

## Abbreviations

AUC, area under curve; CEA, carcinoembryonic antigen; CDDP, cisplatin; ciRNAs, intronic circRNAs; circRNA, circular RNA; ecircRNAs, exonic circRNAs; EIciRNAs, exon–intron circular RNAs; GC, gastric cancer; HuR, human antigen R; IRES, internal ribosome entry site; MBL, Muscleblind protein; miRNA, microRNA; RNase, ribonuclease; RBPs, RNA binding proteins; rRNA, ribosomal RNA; TEM, transmission electron microscopy.
